# Impact of COVID-19 Lockdown on Physical Activity in a Sample of Greek Adults

**DOI:** 10.3390/sports8100139

**Published:** 2020-10-21

**Authors:** Dimitrios I. Bourdas, Emmanouil D. Zacharakis

**Affiliations:** 1 Department of Sports Medicine & Biology of Exercise, National and Kapodistrian University of Athens, Ethnikis Antistasis 41, 17237 Dafni, Greece; 2 School Physical Education and Sport Science, National and Kapodistrian University of Athens, Ethnikis Antistasis 41, 17237 Dafni, Greece; emzach@phed.uoa.gr

**Keywords:** cross-sectional, contagious disease, exercise, MET, public health, quarantine, questionnaire, SARS-CoV-2, sedentary life

## Abstract

It is well known that physical inactivity increases the risk of global death; however, the impact of the coronavirus disease 2019 (COVID-19) lockdown strategy on physical activity (PA) remains unclear. This study compared PA—i.e., daily occupation, transportation to and from daily occupation, leisure time activities, and regular sporting activities—prior (PRE) and during (POST) the on-going COVID-19 outbreak in the Greece lockdown environment. A Greek version of the web-based Active-Q questionnaire was used to access PA. The questionnaire was filled out twice (once each for the PRE and POST conditions) by 8495 participants (age = 37.2 ± 0.2 years (95% confidence interval (CI), 36.9–37.5); males = 38.3% (95%CI, 36.7–40.0); females = 61.7% (95%CI, 60.4–63.0). The relative frequency of overall sporting activities, which, prior to lockdown, occurred at least once per month, and overall participation in competitive sports was significantly reduced (8.6% (95%CI, 7.9–9.3) and 84.7% (95%CI, 82.9–86.6) respectively). With the exception of overall leisure time activities, which were significantly increased in the POST condition, daily occupational, transportation, and sporting activities significant reduced (*p* < 0.05). Overall PA was reduced in all genders, age, body mass index (BMI) and PA level subgroups in the POST condition, and an interaction between the males and High PA subgroups was observed. The change in overall PA (from PRE to POST conditions) was −16.3% (95%CI, −17.3 to −15.4), while in daily occupational, transportation, and sporting activities, it was −52.9% (95%CI, −54.8–51.0), −41.1% (95%CI, −42.8–39.5) and −23.9% (95%CI, −25.1–22.8), respectively. Thus, the lockdown period is highly associated with a negative change in overall PA. During lockdown, inactivity increased dramatically, with males and the high PA population affected significantly more. The decline in PA is a great concern due to possible long-term consequences on public health and healthcare system.

## 1. Introduction

Coronavirus disease 2019 (COVID-19) is an ongoing pandemic [1] and, as a natural consequence of easy airborne contamination, the outbreak has even threatened Greece, with a potential cost in terms of human lives. In the absence of any vaccine intervention or any pharmaceutical treatment (for the time being), the only strategy against the spread of COVID-19 seems to be population separation, reduction of contact, and restriction of movement of people (i.e., quarantine). However, home-based self-quarantine is not a novel measure against contagious diseases. In ancient Greece, the first historical report of restrictive guideline measures to stay at home due to unknown communicable diseases took place in the Attica region, and is dated back to 430 BC, during the Peloponnesian War [2].

COVID-19 first appeared in Greece on 26 February 2020, and the number of confirmed cases as of March 14 was 228 [3]. Following the confirmation of the very first three cases of contamination, health and state authorities initially issued simple precautionary guidelines and recommendations. Many citizens failed to comply, and so gradually, from 13 March and strictly from 23 March to 4 May (41 days overall), lockdown was applied nationwide. Movement outside of the house was permitted only for specific reasons, that including moving to or from the workplace, shopping for food or medicine, visiting a doctor or assisting a person in need for help, and exercising outside individually or in pairs.

The paradox is that while self-isolation and drastic restriction of citizens’ free movement and contact can reduce the risk of virus transmission in the community, at the same time, there is the possibility that the a potential reduction in physical activity (PA) and daily energy expenditure (EE) could create conditions for the development of other non-communicable diseases (NCDs) and an uptick in preventable death rates [4,5,6,7,8], as well as exacerbate behaviors that lead to further physical inactivity and a more sedentary lifestyle, potentially contributing to anxiety, depression, and a deleterious psychological impact [9]. Psychological stress symptoms are increasing as the pandemic continues [9] and are creating imbalances between cortisol and other hormones that have an adverse effect on the immune system and inflammation [10]. On the contrary, PA improves immune function, either after vaccination or in chronic systemic inflammation and various diseases [11,12,13]. Being physically active has significant mental health benefits, reduces symptoms of depression and anxiety (such as in relation to the stress caused by the pandemic), and restores cortisol balance [14]. Moreover, in ≥65-year-old age groups, PA and regular exercise have positive effects on aging characteristics and related diseases, can improve self-esteem and cognitive function, can decrease the risk of falls, and can act to prevent frailty, sarcopenia, and dynapenia [15].

The World Health Organization (WHO) considers PA surveillance an indispensable national public health function [16]. However, currently, there is a lack of empirical data showing that mandated, stay-at-home restrictions affect PA in Greece. Even though we have speculated regarding an increased level of physical inactivity, we do not know the empirical impact of the lockdown on citizens’ PA levels. Additionally, we do not know if gender, age, body mass index (BMI), or PA level (pre-coronavirus outbreak) have an interaction effect. In this sense, a valid assessment of PA is essential for assessing strategies that promote PA, and for surveying and comparing PA levels between different conditions, such us prior to and during the COVID-19 crisis.

Paper questionnaires are commonly used as a tool to assess PA in large epidemiologic studies. Nevertheless, given the threat of coronavirus and the social distancing or self-isolation requirements, as an attempt to keep the disease from spreading by minimizing close contact between individuals, paper questionnaires seem inappropriate for the time being. Alternatively, the use of web-based questionnaires has profound advantages [17]. With this in mind, this study aimed to explore any changes in PA that may have been brought on by the new reality after the “imposition of a temporary restriction on citizens’ traffic in Greece” (lockdown) by using a validated web-based questionnaire. Such data will be of paramount importance in assisting health service plans, determining public health priorities, and preventing inactivity.

## 2. Methods

This study was approved by the National and Kapodistrian University of Athens review board (approval protocol number: 1181/02-04-2020). All participants were given written information about the study and informed consent was provided prior to their voluntary participation. The self-eligibility participation criteria were as follows: (1) aged 18 or above, (2) resident of Greece, and (3) with access to the Internet. Meanwhile, the self-exclusion criteria were as follows: (1) any form of illness, (2) strict weight loss control program participation, and (3) pregnancy or childbirth within one year prior to the start of the study.

### 2.1. Survey Instrument

The revised version of Active-Q, a self-reported, web-based, interactive PA questionnaire assessing habitual activity in adults older than 18 years, was used [18]. Active-Q has been previously validated against doubly labeled water and an accelerometer and has been adequately described elsewhere [18,19]. Prior to the initiation of the study, the original Active-Q (English version) was translated to Greek by two professional translators unaware of the purpose of the study, and then independently back-translated by two technical experts. Any disagreement was resolved by discussion between the translators and technical experts and a consensus was reached. Furthermore, four additional items (i.e., questions) on gender, age, weight, and height were also included [20]. Afterward, Active-Q was freely shared nationwide, and participants were openly invited via social media-hosted links (including public advertisements) or email (snowball distribution strategy).

Overall, the questionnaire comprised 9–46 items, dependent on previous answers and follow-up patterns, designed to assess the frequency and duration of PA across a week. Participants stated their usual PA in four main domains (see Table S1): Daily occupation, transportation to and from daily occupation, leisure time activities, and regular sporting activities. Concisely, all items had a fixed set of answers regarding frequency and duration [18,19] except the questions regarding age, weight, and height. The questionnaire began with a screening question assessing employment status (No/Yes) [18,19], so that for those respondents who stated that they were not working, the upcoming items concerning PA at work were omitted. The opening item in the domains of transportation means, leisure time activities, and sporting activities consisted of screening questions listing all of the activities included in each domain [18,19]. Answers regarding frequency and duration were accepted only for those activities that had been selected by the participants in the screening questions.

### 2.2. Data Collection Procedure

Adults living in Greece were recruited to participate in this study within the period of March to April 2020, which coincided with the very initial stages of the outbreak of COVID-19 and the period of lockdown applied by the authorities. Participants filled out the questionnaire in a double administration fashion at the same time; from here on, in this paper, references to Active-Q will be presume as much (i.e., double administration fashion), unless otherwise stated. The first admission refers to March 2020 (first two weeks; i.e., PA under normal life conditions from 1 to 14 March) prior to the COVID-19 lockdown (PRE condition). The second admission refers to April 2020 (from 4 to 9 April; i.e., PA under lockdown conditions) after the appearance of COVID-19 (POST condition). Data collection took place from 4 to 19 April 2020 (15 days overall). The total time of any PA reported in the Active-Q and in each domain was calculated. All activities were linked to a corresponding metabolic equivalent task (MET) value according to the updated version of the 2011 Compendium of Physical Activities (see Table S1) [21]. Continuously, for each PA duration (min/week), the EE scored in the METs was calculated (e.g., PA of 4 METs × 30 min/day × 2 times per week = 240 MET-min/week). Participants (Table 1) were classified into five ages, four BMI classes PRE condition (Table 2), and four PA categories based on their sport and exercise activities.

### 2.3. Data Analysis

The qualitative variables are reported as frequency or/and relative frequency (%), and quantitative variables as mean ± SE and 95% confidence interval (CI), unless otherwise stated. Chi-squared tests were used to determine whether there was a statistically significant difference in frequency between the PRE and POST conditions, followed by post hoc analysis [22] in cases where a significant difference was found. Independent *t*-tests were used for comparing data between different groups, while dependent *t*-tests were used for comparing PA in the same group under different conditions. One-way ANCOVA was also used to determine whether there were any significant differences in the PA-adjusted means of the POST condition (i.e., adjusted for the PRE condition covariate) between the independent subgroups (by gender, age, BMI classes, or PA levels), followed by post hoc analysis (Bonferroni pairwise comparisons) in cases where a significant difference was found. The level of significance was set at *p* ≤ 0.05 (two-sided) a priori (analyses were done with SPSS for windows v23, IBM Corp., Armonk, NY, USA). Furthermore, because no previous study has been conducted using a similar design, an a priori sample size estimate of >1073 was determined based on a 95%CI, 95% power, and an expected effect size of >0.1 (small) for the mandated, stay-at-home restrictions on PA (estimation was done with G*Power software, version 3.1.9.2, Heinrich-Heine-University Dusseldorf, Dusseldorf, Germany). Moreover, in a preliminary study, the reliability of the Greek version of Active-Q per se recalling data from the recent past (i.e., single administration fashion, two times in separate periods) was tested in 115 subjects. The mean differences between the two admissions were at an acceptable level of 219.4 ± 1450.9 (SD) with a 95%CI according to the Bland Altman plot [23]; the limits of agreement (–2624.4 to 3063.2 = ±1.96 SD) were small enough to consider the Active-Q reliable (see Figure S1).

## 3. Results

In total, 8495 individuals participated in this study. The frequency, the relative frequency (%), and the 95%CI of the participants subgrouped by gender, age, and BMI are presented in Table 2. Table 3 presents the frequency, the relative frequency (%), and the 95%CI of the weekly activity data by PA levels in participants in the PRE and POST conditions. The frequencies in the occupation and transportation activities of the majority of participants were significantly negatively affected by the restriction measures (Table 3). The relative frequencies of overall sporting or exercise activities of participants who PRE had experienced said activity at least once per month were significantly reduced by 8.6% (95%CI, 7.9–9.3) during the quarantine period. Moreover, the relative frequency of overall participation in competitive sports of 16.9% (95%CI, 15.0–18.9) in the PRE condition nearly reached a nadir in the middle of April (2.6% (95%CI, 0.5–4.7)).

**Figure 1:**
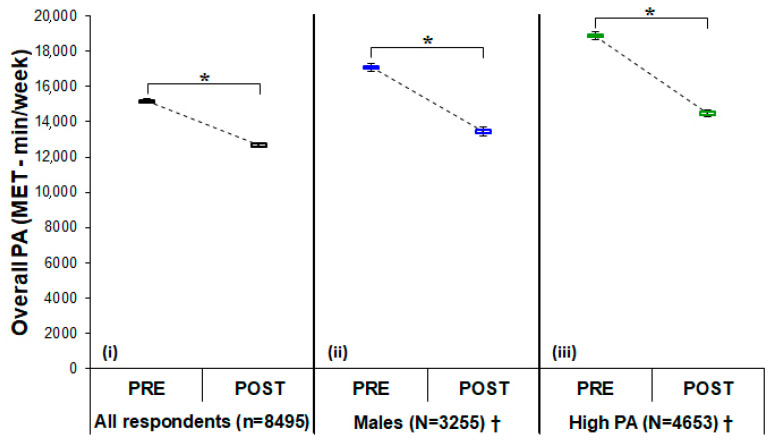
Overall PA in the PRE and POST conditions for all respondents (**i**), males (**ii**), and High PA respondents (**iii**). Data are presented as mean ± SE. * *p* < 0.05, significant difference in all groups between conditions. † *p*< 0.05, significant interaction effect of lockdown on the PA subgroups. Abbreviations: MET, metabolic equivalent task (=3.5 mLO_2_/kg/min); PA, physical activity.

**Figure 2:**
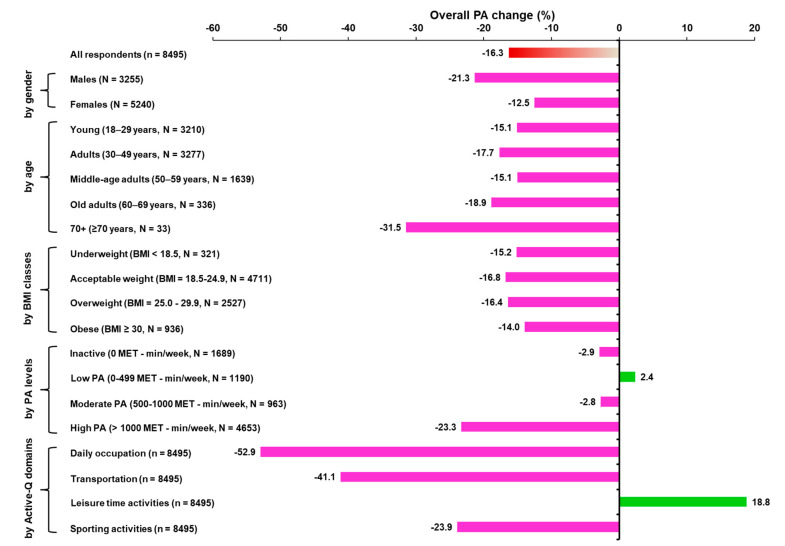
Change in overall PA change (%, from the PRE to POST conditions) on a weekly basis in all respondents, grouped by gender, age, BMI, PA level, and Active-Q domain activities. Abbreviations: BMI, body mass index; MET, metabolic equivalent task (=3.5 mLO_2_/kg/min); PA, physical activity.

The physical activity results in terms of the Active-Q domains and overall PA data from all subgroups in the PRE and POST conditions are presented in Table 4. With the exception of overall leisure time activities, in which EE significantly increased in the POST condition (Table 4), the EE was significantly reduced in the POST condition (i.e., daily occupational, transportation, and sporting activities) (Table 4). The frequency of inactivity significantly increased by 40.6% (95%CI, 38.3–42.9) in the POST condition, while the frequency of moderate and high PA was significantly reduced, by 12.6% (95%CI, 10.5–14.7) and 13.0% (95%CI, 12.0–14.0), respectively (Table 3). In the study population, overall PA was reduced significantly in the POST condition, as well as across the gender, age, BMI and High PA level subgroups (Table 4). Furthermore, the one-way ANCOVA tests revealed that overall PA in the POST condition was significantly affected by gender and PA level. The post hoc analysis revealed a significant interaction between males and the High PA subgroups (Table 5 and Figure 1). The change in overall PA (%, from the PRE to POST conditions) on a weekly basis by all respondents, by all subgroups, and by Active-Q domain activities is depicted in Figure 2. Specifically, the 95%CI of the change in overall PA (from the PRE to POST conditions) was −17.3 to −15.4 in all participants, −23.1 to −19.6 in males, −13.5 to −11.5 in females, and −24.9 to −21.8 in High PA respondents (Figure 2). Moreover, the 95%CI of the change in overall PA (from the PRE to POST conditions) in daily occupational, transportation, and sporting activities was −54.8 to −51.0, −42.8 to −39.5 and −25.1 to −22.8, respectively (Figure 2). The only positive change in overall PA (from the PRE to POST conditions) was observed in leisure time activities (95%CI, 17.9–19.7; Figure 2).

## 4. Discussion 

Though it is well documented that high PA significantly contributes to preventing NCDs and reducing mortality risk factors worldwide, while PA deficiency increases the risk factors of NCDs, it was unknown to what extent lockdown, as a measure against the spread of COVID-19, impaired PA. In our study, it was hypothesized that PA would be negatively affected by the mandated, stay-at-home restrictions. It was found that, indeed, PA changes of −52.9%, −41.1%, and −23.9% occurred in daily occupational, transportation, and sporting activities, respectively, while an excess of leisure time activities, with an 18.8% uptick, was observed. The change in overall PA was −16.3%, which in exercise terms (in this case) corresponds to ~60 min running at an intensity of 8 kmph five times per week [21]. Another finding was the significant interaction effect of lockdown on males and the High PA subgroup on overall PA and a significantly increased frequency by 40.6% of inactivity in the POST condition. 

According to the national housing census revision, Greece’s resident population is 10,816,286 (adults: 80.3%), of which 49.0% are male and 51.0% are female, with a median age range of 30–44 years [24]. The prevalence of overweight and obese people among Greek adults was estimated to be 59.7% and 20.4% for males and 47.9% and 19.9% for females, respectively [25]. Our study group shares similar demographic and anthropometric characteristics, with the exception of the female subgroup, where the prevalence of overweight and obesity appears to be lower in comparison to the WHO 2008 estimates [25].

In the European Union and Greece, 54.0% and 32.0% of people, respectively, tend to practice sports or exercise regularly or even seldomly [26]. In the present study, 73.8% of the participants declared that, prior to the COVID-19 pandemic, they practiced sports or exercise at least once a month, and only 67.5% reported doing so during lockdown. Alongside these findings, a dramatic decrease of 84.7% (95%CI, 82.9–86.6) in the frequency of competition sports activities was observed. Concerning adults’ PA in Greece, the inactivity estimation rose to 41.0% (males 37.0% and females 44.0%) with only 21.0% of the population considered sufficiently active; however, currently, there is a great lack of empirical data demonstrating PA [27,28]. In this regard, the present study is the first to assess the rate of inactivity among Greek residents. In total, the relative frequency of inactivity and low PA prior to COVID-19 were 19.9% and 14.0%, respectively. While these observations seem to situate Greece in the less-sufficient PA countries in the European Union [27], surprisingly, 54.8% of the study population was observed to engage in high PA in terms of sporting activities. This outcome is believed to have resulted from over-reporting PA levels, which was also highlighted as a limitation in a recent relevant Italian study, where the reliable IPAQ questionnaire was used [29]. For that reason, we mainly focused on investigating the change in PA (from the PRE to POST conditions) reported at the same time point.

Similar to our results, a negative change in PA during lockdown was also observed in Australian students and Belgian (<55 years old), Chinese, and Italian people [29,30,31,32,33]. However, most of the relevant studies conducted during lockdown reported that their sample populations were not nationally representative [32,33,34], and since the results of surveys of any country depend on a specific definition of PA that is accepted, along with different sampling methods, statistical modeling, and tools, it is difficult to compare our data with theirs.

As soon as the Greek authorities applied lockdown, the entire population suddenly encountered a new and radical reality. Under these unprecedented conditions, EE during leisure time activities increased. We posit that this is probably a consequence of the greater amount of free time available due to the increased rate of unemployment (22.7%; (95%CI 20.8–24.6)) during lockdown, or perhaps due to the natural body’s need for physical activity against inactivity, for volitional reaction against a sedentary style of life, or for health reasons [26]. Similarly, in two relevant studies in people aged 55+ (years, previously low activity) and body weight lifters, there was a slight increase in PA during lockdown, but it was not clear whether this increment resulted from leisure, sports, or other PA [30,31]. However, in our study, the augmented EE during leisure time activities did not compensate for the overall decremented EE during daily occupational, transportation, or sporting activities due to quarantine. Moreover, although the change in overall PA of −16.3% is an original finding, sadly, this outcome does not comply with the WHO Member States voluntary global target for further reducing the prevalence of insufficient PA by 10.0% by 2025 [35].

Worryingly, our elderly subgroup (70+) significantly changed its overall PA by −31.0% (95%CI, −53.4 to −9.5), but due to the relatively small sample size (*N* = 33), it is hard to draw safe conclusions. Though aging and BMI are related with inactivity and daily EE in a way [26], the age and BMI classes did not show any interaction with PA reduction during lockdown. On the contrary, the overall PA of the male cohort and the High PA subgroup was affected the most and showed a significant interaction with PA in the POST condition. Both males and females are equally motivated to practice sports or to exercise to improve their health [26]. However, gender differences in terms of engaging in sports or other physical activities can be noticed in relation to what motivates people, and perhaps can be derived from gender stereotypes. Males are more likely to engage in sports or physical activities to have fun (33.0%), to be with friends (22.0%), to compete with each other (8.0%), or to improve their physical performance (29.0%), while females are concerned with controlling their weight (24.0%), improving their physical appearance (21.0%), or counteracting the effects of aging (15.0%) [26]. Consequently, as observed in another study [29], the social distancing restriction and the closed sports facilities may have discouraged, to a greater extent, the male subgroup from engaging in sports or physical activities (Table 4). Similarly, the closed sports facilities, the social distancing restrictions, and the absence of any competitive sporting events may have also acted as a deterrent for the High PA subgroup to continue regular high-intensity training.

Lockdown and social distancing were applied to reduce the exposure to and spread of COVID-19 in many countries worldwide [36]. Although the most important goals are to reduce the rate of virus transmission and to develop pharmacological agents, at the same time, we must not underestimate PA and exercise as tools against a sedentary lifestyle [4,5] and for maintaining an adequate health status [7,8]. Physical activity and exercise could also be a powerful combination for overcoming the consequences of the COVID-19 pandemic in many ways, especially for vulnerable groups (e.g., those with underlying conditions, such as diabetes, cardiovascular disease, chronic lung disease, hypertension, cancer, and excess body fat, and for those aged ≥65 years) [37]. 

### Limitations

The findings of this study must be interpreted in the light of some limitations. The first limitation concerns the selection bias derived from the web-based method of data collection. Due to the severity of the current situation, time constraints, and the urgent need to complete the study, we addressed our questionnaire only to the Internet-accessible population. However, fewer than eight out of ten Greek households (78.5%) have Internet access at home [38]. The second limitation is the use of a questionnaire to asses PA as an alternative to a direct measurement (gold standard). We could not have predicted the impending global outbreak of COVID-19, so it was impossible to assess PA beforehand and then to re-assess it after the outbreak. Furthermore, under the COVID-19 pandemic conditions, it was not feasible to measure PA directly in daily life, so we used the Active-Q instead to explore changes in PA from the PRE to POST conditions. Another limitation of this study is that our data revealed consequences of only the two middle weeks out of a total six-week lockdown period and cannot be projected to the full social isolation period. Although we made every effort to use any available social media in order to ensure questionnaire respondents were more representative, a major limitation of this study may be that our sample group is not nationally representative of the adult population. There seems to be an over-representation of female and young female respondents, while the 70+ subgroup of the study was not representative. The PRE PA levels of both sex subgroups also suggest that the sample was skewed toward more physically active younger people, who either had over-reported PA or quite possibly had a relatively higher participation in social media networks and/or a higher socioeconomic status, potentially diminishing the generalizability of the results. However, we believe that this study is the first to clearly demonstrate the impact of lockdown on PA in a sample of Greek adults, as well as the first work in the literature that shows the impact of lockdown on different PAs separately (i.e., daily occupation, transportation to and from daily occupation, leisure time activities, and regular sporting activities). In addition, this study was exploratory and environmental, socioeconomic status, educational level, psychological, or other factors may have, separately or in combination, affected the population’s PA levels, thus generating further hypotheses for future research.

## 5. Conclusions

The Greek measures were among the most proactive and strictest in Europe in an effort to slow down the spread of COVID-19. Nevertheless, the present study empirically demonstrated that the imposition of a temporary lockdown in Greece is highly associated with negative changes in PA in terms of daily occupational, transportation, and sporting activities, with an overall change in PA of −16.3% (95%CI, −17.3 to −15.4). Another important highlight of this study is that during lockdown, inactivity increased overall, yet unequally, with the cohort of male and high PA respondents affected significantly more. Thus, it is obvious that the preexisting PA guidelines from the WHO that were uploaded on some Greek official public health authority sites failed to sufficiently promote overall PA. Furthermore, the decline PA suggests possible long-term consequences on the evolution rate of NCDs, general public health, and healthcare systems, which is a societal concern and requires further research. 

## Figures and Tables

**Table 1:** Anthropometric characteristics of the participants (*n* = 8495).

	Mean ± SE (95% CI)
**Age (years)**	37.2 ± 0.2 (36.9–37.5)
Males	42.1 ± 0.2 (41.6–42.5)
Females	34.2 ± 0.2 (33.8–34.5)
**Height (m)**	171.5 ± 0.1 (171.3–171.7)
Males	179.9 ± 0.1 (179.6–180.1)
Females	166.3 ± 0.1 (166.1–166.5)
**Weight (kg)**	73.0 ± 0.2 (72.7–73.4)
Males	85.6 ± 0.3 (85.1–86.1)
Females	65.2 ± 0.2 (64.9–65.6)
**BMI (kg/m^2^)**	24.7 ± 0.1 (24.6–24.8)
Males	26.4 ± 0.1 (26.3–26.6)
Females	23.6 ± 0.1 (23.5–23.7)

Abbreviations: BMI, body mass index; CI, confidence interval.

**Table 2:** Frequency, relative frequency (%), and 95%CI of participants subgrouped by gender, age, and BMI, (*n* = 8495).

	Frequency (%) (95% CI)
**Gender**	
Males	3255 (38.3) (36.7–40.0)
Females	5240 (61.7) (60.4–63.0)
**Age classes**	
Young (18–29 years)	3210 (37.8) (36.1–39.5)
Adults (30–49 years)	3277 (38.6) (36.9–40.3)
Middle-Age Adults (50–59 years)	1639 (19.3) (17.4–21.2)
Old Adults (60–69 years)	336 (4.0) (1.9–6.1)
70+ (≥70 years)	33 (0.4) (0.0–2.5)
Young (18–29-year-old males)	731 (22.5) (19.4–25.5)
Adults (30–49-year-old males)	1398 (43.0) (40.4–45.5)
Middle-age adults (50–59-year-old males)	874 (26.9) (23.9–29.8)
Old adults (60–69-year-old males)	225 (6.9) (3.6–10.2)
70+ (≥70-year-old males)	27 (0.8) (0.0–4.3)
Young (18–29-year-old females)	2479 (47.3) (45.3–49.3)
Adults (30–49-year-old females)	1879 (35.9) (33.7–38.0)
Middle-age adults (50–59-year-old females)	765 (14.6) (12.1–17.0)
Old adults (60–69-year-old females)	111 (2.1) (0.0–4.8)
70+ (≥70-year-old females)	6 (0.1) (0.0–2.8)
**BMI classes (kg/m^2^)**	
Underweight (BMI <18.5)	321 (3.8) (1.7–5.9)
Acceptable weight (BMI = 18.5–24.9)	4711 (55.5) (54.0–56.9)
Overweight (BMI = 25.0–29.9)	2527 (29.8) (28.0–31.5)
Obese (BMI ≥ 30)	936 (11.0) (9.0–13.0)
Underweight (males)	29 (0.9) (0.0–4.3)
Acceptable weight (males)	1239 (38.1) (35.4–40.8)
Overweight (males)	1487 (45.7) (43.2–48.2)
Obese (males)	500 (15.4) (12.2–18.5)
Underweight (females)	292 (5.6) (2.9–8.2)
Acceptable weight (females)	3472 (66.3) (64.7–67.8)
Overweight (females)	1040 (19.9) (17.4–22.3)
Obese (females)	436 (8.3) (5.7–10.9)

Abbreviations: BMI, body mass index; CI, confidence interval.

**Table 3:** Frequency, relative frequency (%), and 95%CI of the weekly activity data by PA levels in participants in the PRE and POST conditions (*n* = 8495).

PA Levels	PRE Frequency (%) (95% CI)	POST Frequency (%) (95% CI)
Inactive (0 MET-min/week) *	1689 (19.9) (18.0–21.8)	2374 (28.0) (26.1–29.8)
Low PA (0–499 MET-min/week)	1190 (14.0) (12.0–16.0)	1232 (14.5) (12.5–16.5)
Moderate PA (500–1000 MET-min/week) *	963 (11.3) (9.3–13.3)	842 (9.9) (7.9–11.9)
High PA (>1000 MET-min/week) *	4653 (54.8) (53.3–56.2)	4047 (47.6) (46.1–49.2)
Inactive (0 MET-min/week males) *	579 (17.8) (14.7–20.9)	912 (28.0) (25.1–30.9)
Low PA (0–499 MET-min/week males) *	336 (10.3) (7.1–13.6)	420 (12.9) (9.7–16.1)
Moderate PA (500–1000 MET-min/week males)	309 (9.5) (6.2–12.8)	300 (9.2) (6.0–12.5)
High PA (>1000 MET-min/week males) *	2031 (62.4) (60.3–64.5)	1623 (49.9) (47.4–52.3)
Inactive PA (0 MET-min/week females) *	1110 (21.2) (18.8–23.6)	1462 (27.9) (25.6–30.2)
Low PA (0–499 MET-min/week females)	854 (16.3) (13.8–18.8)	812 (15.5) (13.0–18.0)
Moderate PA (500–1000 MET-min/week females) *	654 (12.5) (10.0–15.0)	542 (10.3) (7.8–12.9)
High PA (>1000MET-min/week females) *	2622 (50.0) (48.1–52.0)	2424 (46.3) (44.3–48.2)

* *p* < 0.05, significant difference between PRE and POST conditions. Abbreviations: CI, confidence interval; MET, metabolic equivalent task; PA, physical activity.

**Table 4:** Physical activity data ^a^ by Active-Q domains and overall PA data ^a^ by gender, age, BMI, and PA levels in the PRE and POST conditions, (*n* = 8495).

	PRE (MET-Min/Week)	POST (MET-Min/Week)
**Domains**		
Daily occupation *	4502.7 ± 41.5 (4421.2–4584.1)	2119.4 ± 32.1 (2056.6–2182.3)
Transportation *	1277.7 ± 12.7 (1252.1–1302.0)	751.6 ± 10.7 (730.5–772.7)
Leisure time activities *	6266.6 ± 69.3 (6130.8–6402.3)	7445.7 ± 70.2 (7308.0–7583.3)
Sporting activities *	3114.3 ± 75.5 (2966.2–3262.3)	2369.0 ± 68.8 (2234.2–2503.8)
Daily occupation (males) *	5232.3 ± 72.8 (5089.3–5374.8)	2552.2 ± 58.5 (2437.5–2666.9)
Transportation (males) *	1442.3 ± 22.3 (1398.6–1486.0)	888.3 ± 19.4 (850.3–926.2)
Leisure time activities (males) *	6186.7 ± 117.3 (5956.8–6416.7)	7125.8 ± 118.0 (6894.3–7357.2)
Sporting activities (males) *	4227.1 ± 166.4 (3900.8–4553.4)	2874.4 ± 149.2 (2581.9–3166.8)
Daily occupation (females) *	4049.6 ± 48.9 (3953.8–4145.4)	1850.6 ± 36.7 (1778.7–1922.5)
Transportation (females) *	1174.4 ± 15.1 (1144.7–1204.1)	666.7 ± 12.5 (642.3–691.1)
Leisure time activities (females) *	6316.2 ± 85.5 (6148.6–6483.7)	7644.3 ± 87.0 (7473.9–7814.8)
Sporting activities (females) *	2423.0 ± 63.8 (2298.0–2548.0)	2055.1 ± 61.7 (1934.2–2176.0)
All respondents *	15,160.6 ± 128.6 (14,908.5–15,412.6)	12,685.7 ± 120.0 (12,450.4–12,921.0)
Males *	17,088.2 ± 244.6 (16,608.5–17,567.8)	13,440.6 ± 237.4 (12,975.1–13,906.2)
Females *	13,963.2 ± 140.2 (13,688.3–14,238.0)	12,216.7 ± 126.6 (11,968.6–12,464.8)
**Age classes**		
Young (18–29 years) *	14,406.0 ± 212.3 (13,989.7–14,822.4)	12,230.7 ± 226.3 (11,787.1–12,674.3)
Adults (30–49 years) *	15,668.9 ± 209.7 (15,257.8–16,080.0)	12,894.9 ± 168.0 (12,565.4–13,224.4)
Middle-age adults (50–59 years) *	15,833.7 ± 284.0 (15,276.7–16,390.8)	13,449.1 ± 248.4 (12,961.8–13,936.3)
Old adults (60–69 years) *	14,402.3 ± 547.4 (13,325.5–15,479.8)	11,682.2 ± 596.0 (10,509.8–12,854.6)
70+ (≥70 years) *	12,364.3 ± 1535.2 (9237.1–15,491.5)	8472.6 ± 949.4 (6538.7–10,406.5)
**BMI classes**		
Underweight (BMI < 18.5) *	16,626.9 ± 946.2 (14,765.3–18,488.5)	14,106.3 ± 1053.3 (12,034.0–16,178.7)
Acceptable weight (BMI = 18.5–24.9) *	15,288.2 ± 174.0 (14,947.0–15,629.4)	12,720.7 ± 165.5 (12,396.2–13,045.1)
Overweight (BMI = 25.0–29.9) *	15,022.1 ± 208.9 (14,612.5–15,431.7)	12,552.6 ± 196.4 (12,167.5–12,937.7)
Obese (BMI ≥ 30) *	14,389.2 ± 412.7 (13,579.2–15,199.2)	12,381.8 ± 284.9 (11,822.6–12,941.0)
**PA levels**		
Inactive (0 MET-min/week)	10,792.0 ± 170.7 (10,457.2–11,126.8)	10,477.3 ± 175.6 (10,132.8–10,821.7)
Low PA (0–499 MET-min/week)	10,204.5 ± 179.0 (9853.2–10,555.7)	10,446.0 ± 224.8 (10,005.0–10,887.0)
Moderate PA (500–1000 MET-min/week)	10,993.4 ± 184.2 (10,631.9–11,355.0)	10,691.1 ± 203.8 (10,291.1–11,090.9)
High PA (>1000 MET-min/week) *	18,876.3 ± 202.9 (18478.6–19,274.0)	14,472.9 ± 193.3 (14093.9–14,851.9)

^a^ Data are presented as mean ± SE (95% Confidence Interval). * *p* < 0.05, significant difference between the PRE and POST conditions. Abbreviations: BMI, body mass index; MET, metabolic equivalent task (=3.5 mLO_2_/kg/min); PA, physical activity.

**Table 5:** Overall PA estimates ^a^ in the POST condition by gender, age, BMI and PA levels, adjusted for the PRE condition covariate values (*n* = 8495).

	(MET-Min/Week)
**Gender**	
Males *	12,370.6 ± 157.1 (12,062.7–12,678.6)
Females	12,881.4 ± 123.6 (12,639.1–13,123.6)
**Age classes**	
Young (18–29 years)	12,647.0 ± 157.5 (12,338.2–12,955.8)
Adults (30–49 years)	12,614.5 ± 155.8 (12,309.0–12,919.9)
Middle-age adults (50–59 years)	13,077.7 ± 220.3 (12,645.7–13,509.6)
Old adults (60–69 years)	12,100.5 ± 486.5 (11,146.8–13,054.3)
70+ (≥70 years)	10,015.4 ± 1552.5 (6972.1–13,058.8)
**BMI classes**	
Underweight (BMI < 18.5)	13,296.6 ± 498.0 (12,320.3–14,272.8)
Acceptable weight (BMI = 18.5–24.9)	12,650.2 ± 130.0 (12,395.4–12,905.0)
Overweight (BMI = 25.0–29.9)	12,629.1 ± 177.5 (12,281.2–12,976.9)
Obese (BMI ≥ 30)	12,807.7 ± 291.6 (12,236.0–13,379.4)
**PA levels**	
Inactive (0 MET-min/week)	12,933.2 ± 220.2 (12,501.5–13,364.9)
Low PA (0–499 MET-min/week)	13,232.1 ± 262.0 (12,718.5–13,745.8)
Moderate PA (500–1000 MET-min/week)	13,033.7 ± 289.6 (12,466.1–13,601.3)
High PA (>1000 MET-min/week) *	12,384.1 ± 134.6 (12,120.1–12,648.0)

^a^ Estimates are presented as mean ± SE (95% Confidence Interval). * *p* < 0.05, significant interaction effect of lockdown on PA subgroups. Abbreviations: BMI, body mass index; MET, metabolic equivalent task (=3.5 mLO_2_/kg/min); PA, physical activity.

## Supplementary Materials

The following are available online at https://www.mdpi.com/2075-4663/8/10/139/s1, Table S1: Items included in Active-Q questionnaire by domain activities and corresponding MET values, Figure S1: Difference between PA value of the first and second admission of Active-Q against their mean, (*n* = 115). The mean difference (Δ) is 219.37 ± 1450.91SD with a 95% CI; the limits of agreement (−2624.42–3063.15 = ±1.96SD) are small enough to consider the test reliable. CI = confidence interval; PA = physical activity.
