# Supplementary material for: Impact of COVID-19 Lockdown on Physical Activity in a Sample of Greek Adults
Source: Sports (Basel). 2020 Oct 21;8(10):139. doi: 10.3390/sports8100139 (PMC7589063; doi:10.3390/sports8100139)
Supplement: Supplementary file 1 [file sports-08-00139-s001.zip › sports-963175-supplementary.docx]

Impact of COVID-19 Lockdown on Physical Activity in a Sample of Greek Adults

Dimitrios I. Bourdas ^1,^* and Emmanouil D. Zacharakis ^2^

**Table 1.** Items included in Active-Q questionnaire by domain activities^a^ and corresponding MET values.^1^

| **Domain activities** | **MET value** |
| --- | --- |
| **Daily occupation** |  |
| *Mostly sitting* | 1.5 |
| *A combination of sitting and standing up* | 2.3 |
| *Mostly standing up* | 3 |
| *Some physical activity* | 4.5 |
| *Heavy manual labor* | 6 |
| *Other* | 2.5 |
| **Transportation** |  |
| *Walking* | 4 |
| *Bicycling* | 4 |
| *By motorcycle or scooter* | 2.5 |
| *By car or taxi* | 1 |
| *By bus, train, subway, or boat* | 1 |
| *Other* | 2.5 |
| **Leisure time activities** |  |
| *Watching TV/DVDs* | 1 |
| *Using the computer reading e-mails, playing computer games* | 1 |
| *Sitting listening to music, sewing, etc* | 1 |
| *Playing a musical instrument or active computer games* | 2 |
| *Doing household chores* | 3 |
| *Shopping or other errands* | 2.3 |
| *Dancing* | 3 |
| *Walking (not as transport to daily occupation)* | 3.4 |
| *Bicycling (not as transport to daily occupation)* | 8 |
| *Other* | 2.5 |
| **Sporting activities** |  |
| *Aerobics or cardio fitness class* | 6.5 |
| *Weight lifting* | 6 |
| *Jogging, running or orienteering* | 8 |
| *Athletics, (eg, high jump, long jump or three-step)* | 6 |
| *Spinning or cycling in demanding terrain* | 8.5 |
| *Swimming* | 6 |
| *Martial arts (eg judo or karate)* | 10 |
| *Boxing or wrestling* | 6 |
| *Yoga, Pilates or Tai chi* | 3 |
| *Tennis, badminton or table tennis* | 7 |
| *Squash* | 7.3 |
| *Sailing, surfing, canoeing or rowing* | 3 |
| *Ball sports in team (eg, soccer, basketball, or voleyball)* | 6 |
| *Golf* | 4.5 |
| *Dance class or competitive dancing* | 4.5 |
| *Horseback riding* | 4 |
| *Ice skating, ice hockey or bandy* | 7 |
| *Skiing downhill or cross country* | 7 |
| *Motor sports (eg, motorcross)* | 4 |
| *Rock climbing* | 8 |
| *Other* | 2.5 |

^a^ Daily occupation, transportation, leisure time, and regular sporting activities.

Abbreviations: MET = metabolic equivalent task (=3.5 mLO_2_·kg^−1^·min^−1^);.

Other = Other or Does not apply to me or Don´t know or Don´t want to answer.

Ainsworth, B.E.; Haskell, W.L.; Herrmann, S.D.; Meckes, N.; Bassett, D.R.; Tudor-Locke, C.; Greer, J.L.; Vezina, J.; Whitt-Glover, M.C.; Leon, A.S. 2011 compendium of Physical Activities: A second update of codes and MET values. *Med. Sci. Sports Exerc.* **2011**, *43*, 1575–81.

Recalling data from the past, even if it is only few weeks apart from the present, may contribute bias to the study. Therefore, in a preliminary study the reliability of the Greek version of Active-Q *per se* (i.e., single fashion administration) recalling data from the recent past, was tested in 115 subjects (age: 38 ± 1.17 yr, height: 171 ± 0.86 cm, mass: 73 ± 1.56 kg). The Active-Q administered two times in separate periods at least five days apart each other (28–30 March 2020 and 4–6 April 2020). Both admissions utilized data from the same first two weeks of March 2020 (i.e., PA under normal life conditions). A high correlation coefficient between the two repeated admissions of Active-Q (r = 0.99, [95% CI 0.97–1.00]) was provided. In addition, coefficient of variation (CV) of mean differences (Δ) values between the two series of admissions was ~9%. Mean differences between the two admissions were at an acceptable level of 219.37 ± 1450.91SD with a 95% CI according to the Bland Altman plot;^1^ the limits of agreement (−2624.42–3063.15 = ±1.96SD) were small enough to consider the Active-Q reliable (Figure 1). What's more, participants did not report any difficulties in understanding the questionnaire items or in completing it.


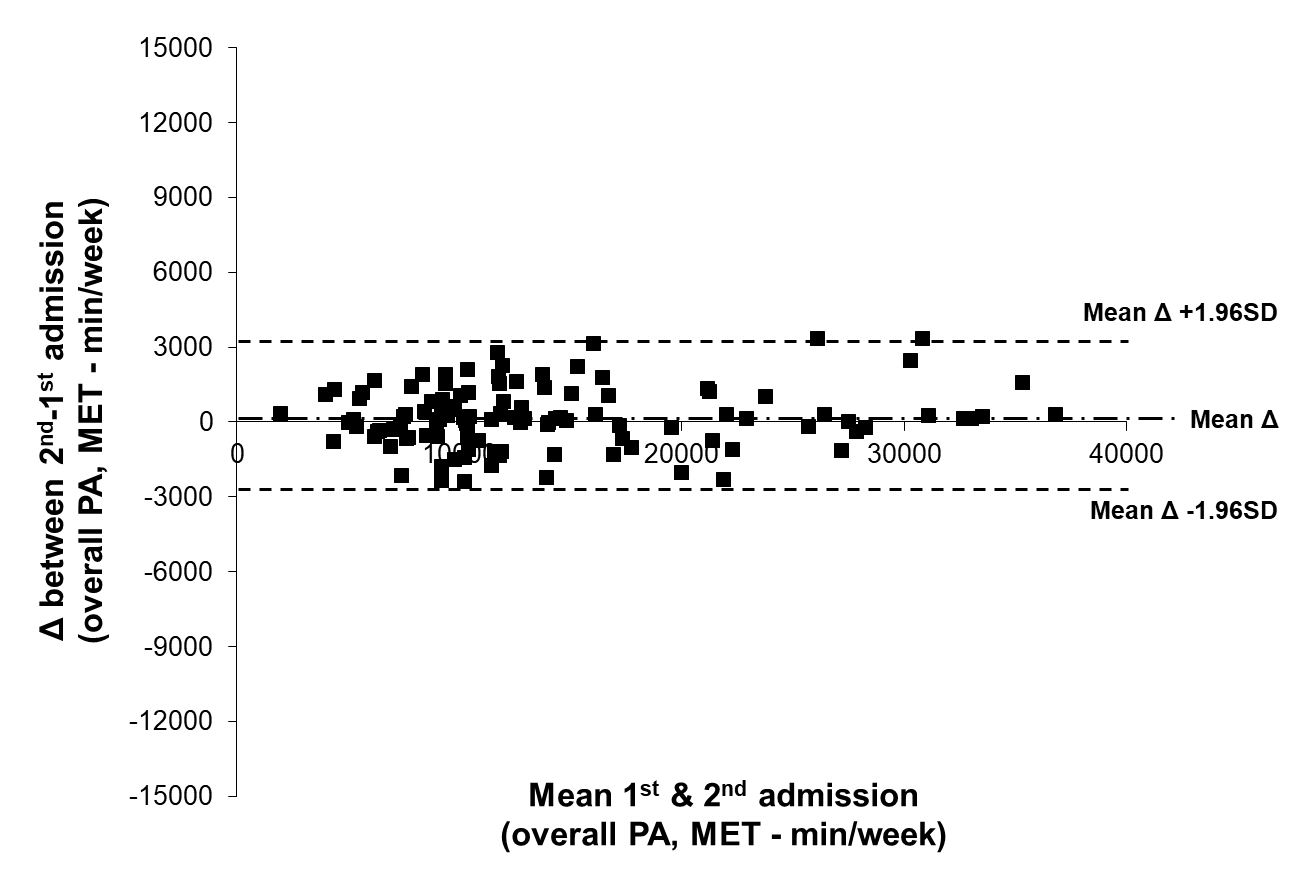


**Figure S1.** Difference between PA value of the first and second admission of Active-Q against their mean, (n = 115). The mean difference (Δ) is 219.37 ± 1450.91SD with a 95% CI; the limits of agreement (−2624.42 – 3063.15 = ±1.96SD) are small enough to consider the test reliable. CI = confidence interval; PA = physical activity.

Bland, J.M.; Altman, D.G. Statistical methods for assessing agreement between two methods of clinical measurement. *Lancet* **1986**, *1*, 307–10.

| 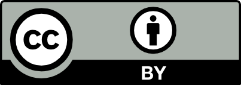 | © 2020 by the authors. Submitted for possible open access publication under the terms and conditions of the Creative Commons Attribution (CC BY) license (http://creativecommons.org/licenses/by/4.0/). |
| --- | --- |
